# Pharmacokinetic and Tissue Distribution of Fucoidan from *Fucus vesiculosus* after Oral Administration to Rats

**DOI:** 10.3390/md16040132

**Published:** 2018-04-18

**Authors:** Olga N. Pozharitskaya, Alexander N. Shikov, Natalya M. Faustova, Ekaterina D. Obluchinskaya, Vera M. Kosman, Heikki Vuorela, Valery G. Makarov

**Affiliations:** 1 Saint-Petersburg Institute of Pharmacy, Leningrad Region, Vsevolozhsky District, Kuzmolovo P 245, 188663 Saint-Petersburg, Russia; olgapozhar@mail.ru (O.N.P.); kosmanvm@mail.ru (V.M.K.); makarov.vg@doclinika.ru (V.G.M.); 2 RMC “House of Pharmacy”, Leningrad Region, Vsevolozhsky District, Zavodskaya str., 3, Kuzmolovo P 245, 188663 Saint-Petersburg, Russia; faustova-78@mail.ru; 3 Federal State Budgetary Scientific Institution of Murmansk Marine Biological Institute, Kola Scientific Center of the Russian Academy of Sciences (MMBI KSC RAS), Vladimirskaya, 17, 183010 Murmansk, Russia; okaterine@yandex.ru; 4 Drug Research Program, Division of Pharmaceutical Biosciences, Faculty of Pharmacy, P.O. Box 56 (Viikinkaari 5E), University of Helsinki, FI-00014 Helsinki, Finland; heikki.vuorela@helsinki.fi

**Keywords:** anti-Xa activity, *Fucus vesiculosus*, fucoidan, kidneys, liver, pharmacokinetic, spleen

## Abstract

*Fucus vesiculosus* L., known as bladderwrack, belongs to the brown seaweeds, which are widely distributed throughout northern Russia, Atlantic shores of Europe, the Baltic Sea, Greenland, the Azores, the Canary Islands, and shores of the Pacific Ocean. Fucoidan is a major fucose-rich sulfated polysaccharide found in *Fucus* (*F.*) *vesiculosus*. The pharmacokinetic profiling of active compounds is essential for drug development and approval. The aim of the study was to evaluate the pharmacokinetics and tissue distribution of fucoidan in rats after a single-dose oral administration. Fucoidan was isolated from *F. vesiculosus*. The method of measuring anti-activated factor X (anti-Xa) activity by amidolytic assay was used to analyze the plasma and tissue concentrations of fucoidan. The tissue distribution of fucoidan after intragastric administration to the rats was characterized, and it exhibited considerable heterogeneity. Fucoidan preferentially accumulates in the kidneys (AUC_0–t_ = 10.74 µg·h/g; C_max_ = 1.23 µg/g after 5 h), spleen (AUC_0–t_ = 6.89 µg·h/g; C_max_ = 0.78 µg/g after 3 h), and liver (AUC_0–t_ = 3.26 µg·h/g; C_max_ = 0.53 µg/g after 2 h) and shows a relatively long absorption time and extended circulation in the blood, with a mean residence time (MRT) = 6.79 h. The outcome of this study provides additional scientific data for traditional use of fucoidan-containing plants and offers tangible support for the continued development of new effective pharmaceuticals using fucoidan.

## 1. Introduction

*Fucus vesiculosus* L., known as bladderwrack, belongs to the brown seaweeds, which are widely distributed throughout northern Russia, Atlantic shores of Europe, the Baltic Sea, Greenland, the Azores, the Canary Islands, and shores of the Pacific Ocean. Many species of brown seaweeds have been used in food products and also documented as being used in traditional medicine [1,2]. Some Western herbal products containing *F. vesiculosus* are known to be used topically for the treatment of sore knees [3], healing wounds [4], and also as herbal teas for their laxative or weight control effects [5]. *F. vesiculosus* has been reported for the treatment of the uterus and ovaries in the Caribbean islands [6].

Fucoidan is a cell-wall fucose-rich sulfated polysaccharide that is mainly found in brown algae of the class Phaeophyceae [7]. Fucoidan has been used as an anticancer drug in traditional Chinese medicine [8]. It shows a wide range of biological activities including anticoagulant, anti-inflammatory [9], antidiabetic [10], procoagulant [11], anticancer [12], and antiviral activities [13]. Currently, fucoidan is not approved for medical applications. However, it is considered a promising drug candidate due to its low toxicity [14], good biocompatibility [15], and encouraging results in preclinical and sporadic early-stage clinical trials [16,17].

The pharmacokinetic profiling of active molecules is essential for the drug development process. Reliable analytical methods are required for the understanding of the pharmacokinetics of fucoidan. A limited number of assays for the quantification of fucoidan in plasma or biological samples have been reported. A competitive ELISA was developed and used for the detection of *Undaria (U.) pinnatifida* fucoidan in plasma after oral ingestion of the compound by human volunteers [18]. Later, a more sensitive sandwich ELISA was described for *Cladosiphon okanuramus* fucoidan in human serum and urine [19]. Methods using electrochemical detection [20] and direct fluorescent assay of brown algae-derived fucoidans in human plasma with the commercially available Heparin Red Kit [21] have been reported.

Heparins display certain structural similarities to fucoidans with respect to their polysaccharide nature and high negative charge density due to sulfation. A positive correlation between biological activity and concentration in plasma was demonstrated for heparin [22,23]. A recent report suggested that plasma antiactivated factor X (anti-Xa) activity must be tested in order to achieve an effective anticoagulant level in patients receiving anticoagulant therapy with enoxaparin [24]. An anti-Xa chromogenic assay was validated for the quantification of enoxaparin in human plasma [25].

Some authors have proposed that fucoidan shows heparin-like actions such as prolonging activated partial thromboplastin time via anti-Xa and anti-IIa activity [16]. However, we have found no reports regarding the tissue distribution of fucoidan after oral administration. In our study, a method for detecting fucoidan based on its anti-Xa activity was established in rats. The method was used to investigate the pharmacokinetic and tissue distribution of fucoidan isolated from *F. vesiculosus* after a single-dose oral administration.

## 2. Results

### 2.1. Chemical Composition of Fucoidan

The average molecular mass of the purified fucoidan was estimated to be 735 kDa. The polysaccharide contained 79.5% neutral carbohydrates, 27.0% sulfate residues, and 0.7% uronic acid. According to the HPLC analysis, the fucoidan contained fucose, glucose, galactose, xylose, mannose, and arabinose at a molar ratio of 1.0:0.16:0.05:0.09:0.03:0.03, respectively. The major monosaccharide was fucose (73.5 mol%), followed by glucose (11.8 mol%) and xylose (6.6 mol%). Other monosaccharides were present at minor concentrations.

### 2.2. Method Validation

The International Conference on Harmonization (ICH) guidelines on the validation of analytical methods were used [26,27]. A comparison of the UV spectra of the reaction products showed that the components in blood plasma do not affect the spectra of the reaction products of the heparin kit. The calibration curve for fucoidan was linear over a concentration range of 0.027–0.217 μg/mL. The fucoidan concentration was calculated according to the equation: *у* = 0.1234*x* + 0.0197; (R^2^ = 0.992), where *у* is the concentration (μg/mL) and *х* is the anti-Xa activity from which the endogenous level was subtracted. The validation data for the method of determining fucoidan concentration in blood plasma are presented in Table 1.

### 2.3. Pharmacokinetic and Tissue Distribution

**Figure 1:**
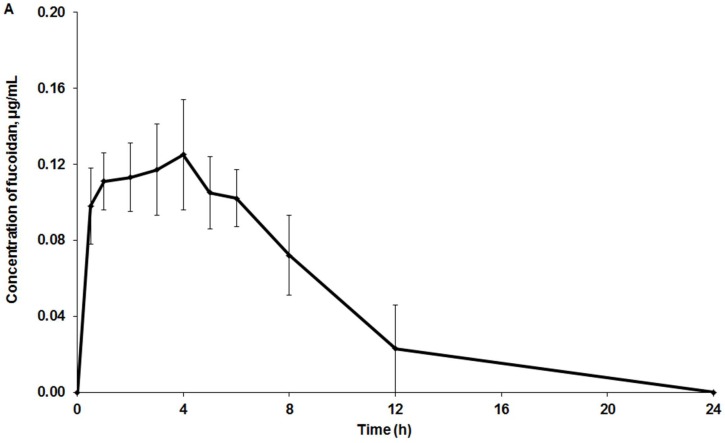

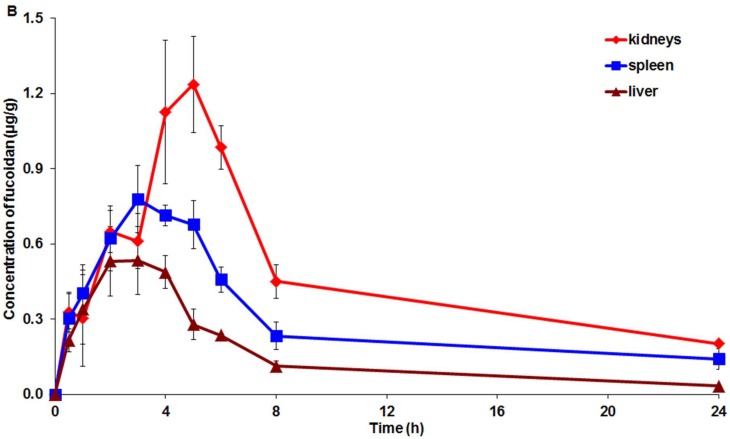
In vivo distribution of fucoidan after intragastric administration to rats. Mean (± standard deviation) drug concentrations in plasma (**A**), and kidneys, spleen, and liver (**B**) (*n* = 5 measurements per time point).

No such clinical signs of toxicity as mortality, aggression, changes in locomotor activity, tremor, convulsions, pain, or touch response were observed in the treated group. Figure 1 shows the mean plasma and tissues profiles of fucoidan after intragastric administration to the rats.

The highest concentration of fucoidan (C_max_ = 1.23 µg/g) was found in the kidneys, while the lowest level was found in the plasma (C_max_ = 0.125 µg/mL, Figure 1). The pharmacokinetic parameters of fucoidan distribution in plasma, kidneys, spleen, liver, striated muscle, and omentum are presented in Table 2. The values of tissue availability (f_t_) provided evidence about the highest concentration of fucoidan in the kidneys.

## 3. Discussion

The pharmacokinetics and tissue distribution are crucial in understanding biological activity. The microdetermination of fucoidan distribution is one of the key problems in its pharmacokinetic studies. Natural polysaccharides contain very few chromophores and reactive groups. The analysis of fucoidan in biological samples is complicated because of its metabolites and its low concentration in vivo after oral administration. It has been proposed that the various biological effects of fucoidan originate in the physiological reactions in the gastrointestinal tract and that fucoidan is scarcely absorbed into the blood [19]. We believe that this opinion has been formed because there have been no sensitive methods to detect fucoidan in biological samples.

In our study, an anti-Xa activity assay for the detection of fucoidan was successful in the characterization of the pharmacokinetics and tissue distribution of fucoidan in plasma and tissues.

The results of this study demonstrate that fucoidan at the dose of 100 mg/kg with molecular weight of 735 kDa was detected in rat plasma 30 min after the intragastric administration. The C_max_ = 0.125 µg/mL was observed at 4 h (Figure 1). The highest concentration of fucoidan (66 kDa) after a single oral administration of 1 g in human volunteers (63 ng/mL) was observed by ELISA in serum at 6 or 9 h [19]. The doses of fucoidan in our study and that of Tokita et al. [19] were similar, taking into account the human equivalent dose (HED). However, the fucoidan concentration in the human blood was about two times lower than the level obtained by us. Apparently, this finding results from a faster rate of metabolism in rats and the different source of fucoidan and its molecular weight. An ELISA competitive-based assay was used for the analysis of fucoidan in plasma from healthy volunteers who for 12 days, ingested 3 g/day of extracts from *U. pinnatifida* containing 10% or 75% of fucoidan (713 kDa) [18]. The plasma concentration in samples from volunteers who received 10% or 75% fucoidan was 4.00 or 12.99 mg/L, respectively, while the highest concentration after 12 days was 5.08 or 15.75 mg/L, respectively. The concentration of fucoidan in our study was much lower than the levels obtained by Irhimeh et al. [18]. This difference may be explained by the cumulative effect and higher doses of fucoidan used in the human trial, differences in metabolism, and analytical methods used. Recently, Zhang et al. [28] reported a HPLC method with postcolumn fluorescence derivatization for the analysis of fucoidan and its metabolites in rabbits. The limit of detection and limit of quantification were determined to be 0.125 μg/mL and 0.5 μg/mL, respectively. Using this method, it was shown that low-molecular-weight (7.1 kDa) fucoidan (50 mg/kg of body weight, intravenous administration) was rapidly absorbed in rabbits. The highest concentration of 110.53 μg/mL was observed at 5 min. The concentration–time curve was biexponential, while the half-life times were T_1/2α_ = 11.24 ± 2.93 min and T_1/2β_ = 98.20 ± 25.78 min. No drug was detected after 6 h. However, the sensitivity of the method was too low for the qualitative analysis of fucoidan after intragastric administration to rabbits (200 mg/kg of body weight). The drug was detected only approximately 2 h after administration, but no concentration was provided in the manuscript. We suppose that the sensitivity of that method was lower than our ELISA method. However, the pretreatment of biological samples was also different and may play a critical role as well. Intestinal absorption of fucoidan (28.8 kDa) was studied in rats fed 2% fucoidan chow for one or two weeks. Serum fucoidan reached a concentration of 2.7 ng/mL after two weeks. According to histological data, the epithelial cells of the jejunum were stained positively for fucoidan [29].

The low-molecular-weight fucoidan was quite quickly eliminated from the blood after intravenous injection in rabbits (MRT = 109 min) and was not detected in the blood 2 h after intragastric administration [28]. Our results imply that intragastric administration of high-molecular-weight fucoidan gave the prolonged MRT value of 6.79 ± 1.63 h and provided evidence about the relatively low absorption and long circulation of fucoidan in the blood.

Furthermore, our study is the first study in which the tissue distribution of fucoidan in rats after a single-dose oral administration has been studied. We found that fucoidan preferentially accumulates in the organs with the greatest “filtering” function, namely the kidneys, spleen, and liver (Table 2). In our study, fucoidan was mainly concentrated in the kidney. Recently, the renoprotective role of fucoidan was confirmed in animal studies using models of kidney injury [30,31]. This finding agrees with our evidence of the relatively long MRT of 13.39 h in the kidney.

High-molecular-weight fucoidan enhances the viability and prevents the death of spleen cells [32]. Interestingly, in our experiments, the most prolonged MRT of 14.57 h was observed for fucoidan in the spleen (Table 2). Administration of fucoidan reduced CCl_4_-induced acute and chronic liver failure in rats [33]. Our findings regarding the liver distribution of fucoidan support and could explain this effect as well. Chen et al. [34] have reported that fucoidan supplementation improves exercise performance and exhibit antifatigue actions in mice. In our study, fucoidan was determined in the muscles for the first time. The AUC of fucoidan was relatively low; however, its antifatigue function may be associated with its MRT of 5.43 h.

## 4. Materials and Methods

### 4.1. Materials

The brown seaweed *F. vesiculosus* was randomly collected from the littoral of the Barents Sea (Dalnie Zelentsy, Murmansk region, Russia) in August 2015. Seaweed was washed with fresh water, frozen, and stored at −18 °C before being analyzed. The identification was performed by Dr. E.D. Obluchinskaya. The voucher specimen (No. 8.2015, V.D.Z.) was deposited in the Collection of the Algology Laboratory, Murmansk Marine Biology Institute. Analytical grade chemicals and solvents for extraction and analysis were purchased from local chemical suppliers. Deionized water, filtered through a 0.22-μm filter, was used to reconstitute the calibrators, factor Xa, chromogenic substrate, and antithrombin.

### 4.2. Extraction Procedures

Fucoidan was isolated as earlier shown [35], with some modifications. Briefly, a frozen seaweed sample (100 g) was ground and extracted with 100 mL of a mixture of methylene chloride/ethanol (94.2:5.8, *v*/*v*). After filtration, the residue was extracted in an ultrasound bath (Branson 3510DTH, Branson Ultrasonics Corp., Danbury, CT, USA) with a 5% aqueous solution of ethanol at 40 °C for 4 h at pH 3–4. The liquid fraction was isolated by centrifugation (10,000× *g* for 15 min) using Sigma 2K15 (Mason Technology, Dublin, Ireland). After centrifugation, the crude fucoidan was dialyzed through a tangential membrane filter and freeze-dried. The yield was 4.9% by mass.

### 4.3. Analysis of Fucoidan Composition

For the analysis of the molecular weight distribution, fucoidan was dissolved in highly purified H_2_O, filtered through a membrane filter (0.45 μm), and separated over Shodex Asahipak (Kanagawa, Japan) GS-520 HQ and GS-620 HQ (7.5 × 300 mm) columns. The elution was done by H_2_O (0.5 mL/min) at 60 °C. Standard pullulans with MWs 6.2–740 kDa (Polymer Laboratories, Houston, TX, USA) were used for calibration of columns. The molecular mass distribution of fucoidan was assessed by normalizing the peak areas [36]. The total neutral carbohydrate of fucoidan was analyzed by the phenol-sulfuric acid method [37] using l-fucose as a reference. The BaCl_2_-gelatin method was used for sulfate residue determination [38], using Na_2_SO_4_ as a standard. Uronic acid content was analyzed by the carbazole reaction [39], using d-glucuronic acid as a reference.

The monosaccharides were analyzed by high-performance liquid chromatography (Shimadzu HPLC system, Kyoto, Japan) according to a previous method [40].

### 4.4. Animals

Male rats (*n* = 50) were obtained from Rapplovo animal house (St. Petersburg, Russia). The animals were kept under standard conditions with a 12-h light–dark cycle, at ambient temperature (22 ± 2 °C), and relative humidity of 60 ± 10%. They had free access to food (Standard diet: Volosovo, Russia) and water ad libitum. Rats (*n* = 5 per time point) were fasted overnight before the experiment. In human experiments, fucoidan was safe at the oral dose of 1 g/day [3,19]. It was shown in our preliminary study that fucoidan was not toxic after intragastric administration to rats at doses up to 2000 mg/kg. The fucoidan dose was converted from a human equivalent dose (HED) by the following formula recommended by the U.S. Food and Drug Administration [41]. The HED (human weight of 60 kg) for 1 (g)/60 (kg) = 0.0167 × 6.2 = a rat dose of 0.10354 g/kg or ~100 mg/kg. The rationality of this dose selection is supported by previous reports. Renoprotective activity of fucoidan was observed at the dose of 100–200 mg/kg/day in Wistar rats [30]. A dose of 100 mg/kg/day was effective in streptozotocin-induced diabetic rats [10]. Fucoidan (100 mg/kg) in starch slime was administered to rats intragastrically by gavage. After administration, the rats were euthanized in a CO_2_ chamber and the blood was collected in sodium citrate tubes by cardiac puncture at different time points (30 min, 1, 2, 3, 4, 5, 6, 8, 12, and 24 h). The blood was centrifuged at 3000× *g* for 15 min at 4 °C, then the plasma was collected and stored at −20 °C. The organs were removed by surgical resection. The organs with different vascularity were selected according to the recommendations [42].

Each organ sample was precisely weighed and homogenized (Polytron PT-MR 1600E, Kinematica AG, Lucerne, Switzerland) in 0.15 μM Tris-HCl buffer (pH 8.4). After vortex-mixing and centrifugation for 15 min at 3000× *g* (EBA21 tabletop centrifuge, Hettich, Westphalia, Germany), the upper phase was collected and used for an amidolytic assay.

Experiments were performed according to the directive 267, “Regarding the statement of regulation of laboratory practice of the Ministry of Health of the Russia” (2003) and the EEC Directive of 1986 (86/609/EEC), and were approved by the Ethical Commission of the St. Petersburg Institute of Pharmacy (Leningrad Region, Vsevolozhsky District, Kuzmolovo P 245, Russia).

### 4.5. Analysis of Anti-Xa Activity

The anti-Xa activity was analyzed by amidolytic assay using a ReaChrom Heparin kit (Renam, Russia). The reaction sequence underpinning the anti-Xa assay includes three steps (adopted from [25]):

(1) fucoidan + antitrombine III (ATIII) → ATIII–fucoidan
(2) ATIII–fucoidan + Xa → ATIII–fucoidan–Xa + Xa_free_
(3) Xa_free_ + substrate–pNA → peptide + pNA

Steps (1) and (2) occur in a plasma/organ sample. Step (3) occurs when the substrate is added to the plasma/organ sample in the laboratory test. Factor Xa is not inactivated by the fucoidan and is free to react with the added substrate, resulting in the formation of p-nitroanilide (pNA). The appearance of pNA, determined spectrophotometrically, is inversely proportional to the concentration of fucoidan in a plasma/organ sample.

The test solutions were prepared by mixing 20 μL of an organ homogenate or calibration standards of fucoidan or a calibrator solution with an appropriate volume of 0.15 μM Tris-HCl buffer (pH 8.4) to fit with concentration in a linear range, while plasma samples were used without dilution. The appropriate dilution coefficient was used for calculations.

The reaction mixture consisting of 20 μL of the test solution, 20 μL of an ATIII solution (activity of 0.2 U/mL), and 80 μL of 0.15 μM Tris-HCl buffer was incubated (ST-3L shaker, Elmi, Latvia) at 37 °C for 2 min. Then, 40 μL of aqueous solution of factor Xa (activity of 2 U/mL) was added for the initiation of reaction. After incubation at 37 °C for 5 min, 40 μL of a synthetic chromogenic substrate (2 mM) was added. Five minutes later, the reaction was stopped by adding of 80 μL of 50% acetic acid. A control contained 60 μL of buffer instead of the test solution and factor Xa. The optical density of free pNA was determined at 405 nm using a microplate spectrophotometer (X-Mark; Bio-Rad, Hercules, CA, USA). The endogenous level of products reacted with the heparin kit was subtracted at each time point in each sample.

### 4.6. Pharmacokinetic and Statistical Analysis

The pharmacokinetic calculations for fucoidan in organs and plasma were performed with the Excel add-in program PK Solver. The parameters were calculated from the concentration-time data using a noncompartmental pharmacokinetic model as described previously [43]. The results are expressed as the mean ± standard deviation (*n* = 5 for each time point). Statistica ver. 6.0 software (Statsoft, Palo Alto, CA, USA) was used for analysis.

## 5. Conclusions

This report is the first study of fucoidan to reveal its important fundamental pharmacokinetic properties and tissue distribution after oral administration to rats. The outcomes of this study provide additional scientific data for the traditional use of fucoidan-containing plants and offers tangible support for the continued development of new effective pharmaceuticals containing fucoidan.

## Figures and Tables

**Table 1:** The validation data for the method of determining fucoidan in blood plasma.

Parameter	Range
Accuracy, %	
ULOQ (0.217 μg/mL)	0.64–3.10
Middle-quality control (0.108 μg/mL)	1.12–4.20
Low-quality control (0.054 μg/mL)	5.5–12.0
LLOQ (0.027 μg/mL)	3.0–7.1
Intraday//Interday precision (RSD), %	
ULOQ (0.217 μg/mL)	0.8–2.5//2.3
Middle-quality control (0.108 μg/mL)	4.0–4.5//4.6
Low-quality control (0.054 μg/mL)	6.5–8.1//8.6
LLOQ (0.027 μg/mL)	0.7–6.2//11.6
LOD, μg/mL	0.01

ULOQ, upper limit of quantification; LLOQ, lower limit of quantification; LOD, limit of detection.

**Table 2:** Pharmacokinetic parameters of fucoidan in plasma and tissues after intragastric administration to rats.

Sample	Parameters			
	AUC_0–t_ (µg·h/g) *	MRT (h)	T_1/2_ (h)	f_t_
Plasma	0.99 ± 0.27	6.79 ± 1.63	3.44 ± 1.70	-
Liver	3.26 ± 1.54	9.25 ± 3.78	6.44 ± 3.57	3.29
Kidneys	10.74 ± 5.15	12.39 ± 4.26	7.26 ± 3.09	10.85
Spleen	6.89 ± 2.87	14.57 ± 6.51	9.32 ± 5.12	6.96
Striated muscle	1.49 ± 0.22	5.43 ± 0.82	2.36 ± 0.84	1.50
Omentum	1.10 ± 0.22	7.78 ± 0.93	4.30 ± 0.79	1.11

* AUC_0–t_ (μg·h/mL) for plasma. AUC_0–t_, the area under the curve; MRT, mean residence time; T_1/2_, apparent half-life of elimination; f_t_, the tissue availability. The results are expressed as the mean ± SD (*n* = 5).
